# Giving Your Electronic Health Record a Checkup After COVID-19: A Practical Framework for Reviewing Clinical Decision Support in Light of the Telemedicine Expansion

**DOI:** 10.2196/21712

**Published:** 2021-01-27

**Authors:** Jonah Feldman, Adam Szerencsy, Devin Mann, Jonathan Austrian, Ulka Kothari, Hye Heo, Sam Barzideh, Maureen Hickey, Catherine Snapp, Rod Aminian, Lauren Jones, Paul Testa

**Affiliations:** 1 Medical Center Information Technology NYU Langone Health New York, NY United States; 2 Department of Medicine NYU Long Island School of Medicine Mineola, NY United States; 3 Department of Medicine NYU Grossman School of Medicine New York, NY United States; 4 Department of Population Health NYU Grossman School of Medicine New York, NY United States; 5 Department of Pediatrics NYU Long Island School of Medicine Mineola, NY United States; 6 Department of Obstetrics and Gynecology NYU Long Island School of Medicine Mineola, NY United States; 7 Department of Orthopedics NYU Long Island School of Medicine Mineola, NY United States

**Keywords:** COVID-19, EHR, clinical decision support, telemedicine, ambulatory care, electronic health record, framework, implementation

## Abstract

**Background:**

The transformation of health care during COVID-19, with the rapid expansion of telemedicine visits, presents new challenges to chronic care and preventive health providers. Clinical decision support (CDS) is critically important to chronic care providers, and CDS malfunction is common during times of change. It is essential to regularly reassess an organization's ambulatory CDS program to maintain care quality. This is especially true after an immense change, like the COVID-19 telemedicine expansion.

**Objective:**

Our objective is to reassess the ambulatory CDS program at a large academic medical center in light of telemedicine's expansion in response to the COVID-19 pandemic.

**Methods:**

Our clinical informatics team devised a practical framework for an intrapandemic ambulatory CDS assessment focused on the impact of the telemedicine expansion. This assessment began with a quantitative analysis comparing CDS alert performance in the context of in-person and telemedicine visits. Board-certified physician informaticists then completed a formal workflow review of alerts with inferior performance in telemedicine visits. Informaticists then reported on themes and optimization opportunities through the existing CDS governance structure.

**Results:**

Our assessment revealed that 10 of our top 40 alerts by volume were not firing as expected in telemedicine visits. In 3 of the top 5 alerts, providers were significantly less likely to take action in telemedicine when compared to office visits. Cumulatively, alerts in telemedicine encounters had an action taken rate of 5.3% (3257/64,938) compared to 8.3% (19,427/233,636) for office visits. Observations from a clinical informaticist workflow review included the following: (1) Telemedicine visits have different workflows than office visits. Some alerts developed for the office were not appearing at the optimal time in the telemedicine workflow. (2) Missing clinical data is a common reason for the decreased alert firing seen in telemedicine visits. (3) Remote patient monitoring and patient-reported clinical data entered through the portal could replace data collection usually completed in the office by a medical assistant or registered nurse.

**Conclusions:**

In a large academic medical center at the pandemic epicenter, an intrapandemic ambulatory CDS assessment revealed clinically significant CDS malfunctions that highlight the importance of reassessing ambulatory CDS performance after the telemedicine expansion.

## Introduction

The COVID-19 pandemic has ushered in seismic changes in the delivery of care, as telemedicine has revolutionized and likely permanently altered how outpatient care is delivered [1,2]. Telemedicine is not just office medicine virtualized; rather, there are dramatic differences in workflows [3], differences in the composition of and interaction between members of the care team, and differences in the type and quality of clinical data available to clinicians at the time of the telemedicine encounter. With this shift, some unintended consequences for providing preventive and chronic care have been documented [4-7]. The need for rapid transition from ambulatory in-person visits to telemedicine encounters, confounded by limited resources as a byproduct of the pandemic, has further magnified chronic care management challenges.

When properly deployed, clinical decision support (CDS) tools ensure that the right information is presented in the appropriate workflow to support clinical decision making. However, two-thirds of chief medical information officers report at least 1 CDS malfunction annually [8], and a study of electronic health record (EHR) alerts at a leading academic medical center revealed that 22% of active alerts were broken [9]. Ongoing evaluation of an organization's CDS program is critical to advance patient safety, quality, and experience of care [10,11]. As stewards of hard-earned successes in CDS-driven health care improvement, informaticists are responsible for remaining vigilant in supporting CDS-driven general health, well-being, and chronic conditions management. This is perhaps even more important during the pandemic, when our CDS is at higher risk of malfunctioning, and when these aspects of care are at risk of being neglected [12]. Due to practicing medicine during the pandemic, significant competing priorities by necessity force us to employ a time-sparing and straightforward approach to evaluate the health of our outpatient CDS program in the context of the COVID-19 telemedicine expansion.

## Methods

NYU Langone Health (NYULH) is a large academic health care system in New York, consisting of over 5000 health care providers across 4 hospitals and ≥500 ambulatory locations. Since 2011, NYULH has grown its ambulatory care network across Manhattan, Brooklyn, Queens, Staten Island, Long Island, and Florida, and has maintained its position as a national leader in high-quality outpatient care, receiving the Ambulatory Care Quality and Accountability Award from Vizient Inc in each of the past 4 years. In numerous ways, NYULH's implementation of a single EHR (Epic Systems) and integration of ancillary systems help to facilitate ongoing excellence in ambulatory quality by connecting the vast network of locations, supporting best practice with electronic decision support and presenting dashboards that reinforce the NYULH culture of data-driven performance and accountability. This organizational structure provides the ideal context to assess ambulatory CDS for chronic disease.

In this report, our study period is March 19 to May 31, 2020, a time frame representing the start of the COVID-19 pandemic–related telemedicine expansion up until the end of May. Throughout this time, NYULH had been at the epicenter of the first wave of the national COVID-19 pandemic. NYULH consolidated outpatient practices and redeployed ambulatory providers to the inpatient setting. In-person office visits continued, but most patients opted for telemedicine video visits with their usual ambulatory care providers. During the study period, 2100 providers completed 244,425 telemedicine video visits. These video visits accounted for 59% (244,425/414,076) of the ambulatory visit volume, with in-person office visits accounting for the other 41% (169,651/414,076).

To evaluate how this shift toward telemedicine impacted ambulatory CDS at NYULH, our clinical informatics team developed a basic framework for assessing our CDS program's fitness to navigate the transformation. The framework included the following 4 steps:

1. Analysis of alert firing volumes and per-encounter firing rates in telemedicine encounters and office visits.
2. Analysis of action taken rates for the same alerts shown in telemedicine encounters and office visits.
3. Clinical informaticist review of alerts with significant discrepancies in firing volume or action taken rates using the 5 Rights of CDS to identify optimization opportunities.
4. Review of optimization opportunities through the existing CDS governance structures and consideration of ways to enhance CDS governance for rapid transformation.

Our framework builds upon previously published work [13] that describes alert malfunction as occurring across two major domains: (1) malfunction in alert display and (2) malfunction in provider response. We chose firing volumes and per-encounter firing rates to assess for dysfunction in CDS alert display. Firing rates for the same alert may vary significantly across clinical care settings [14]. We aimed to understand if telemedicine as a care setting demonstrated significantly different firing volumes or firing rates than office visits. Regular review of alert firing rates is the best practice for identifying alert display malfunctions [15]. Many organizations, like ours, have adopted dashboards for ongoing monitoring of alert firing rates and firing volumes [16-18]. Though we have existing dashboards, because of the comparative nature of our approach (comparing behavior between telemedicine and office visits), for this evaluation we used the Epic system’s Slicer Dicer BPA data model for data extraction.

As the second domain of alert malfunction, we looked at clinician response. Though there are many ways to measure alert performance in this domain [19,20], we chose the action taken rate, defined as the rate at which a clinician takes any action toward acknowledging a displayed alert. This measure allowed us to look for trends across many alerts with different action types. We also sought to understand whether the action taken rates for the same alert differed across telemedicine and office visits. At NYULH, providers experience the same user interface with the same activities and navigators in telemedicine and office visits. Thus, differences in the alert action rates represent actual disparities in the CDS of a single alert presented in two different clinical contexts. Again, we used the Epic system’s Slicer Dicer BPA data model for data extraction and group comparison.

## Results

### Evaluating Ambulatory Alert Firing Volumes After the COVID-19 Telemedicine Expansion

**Figure 1:**
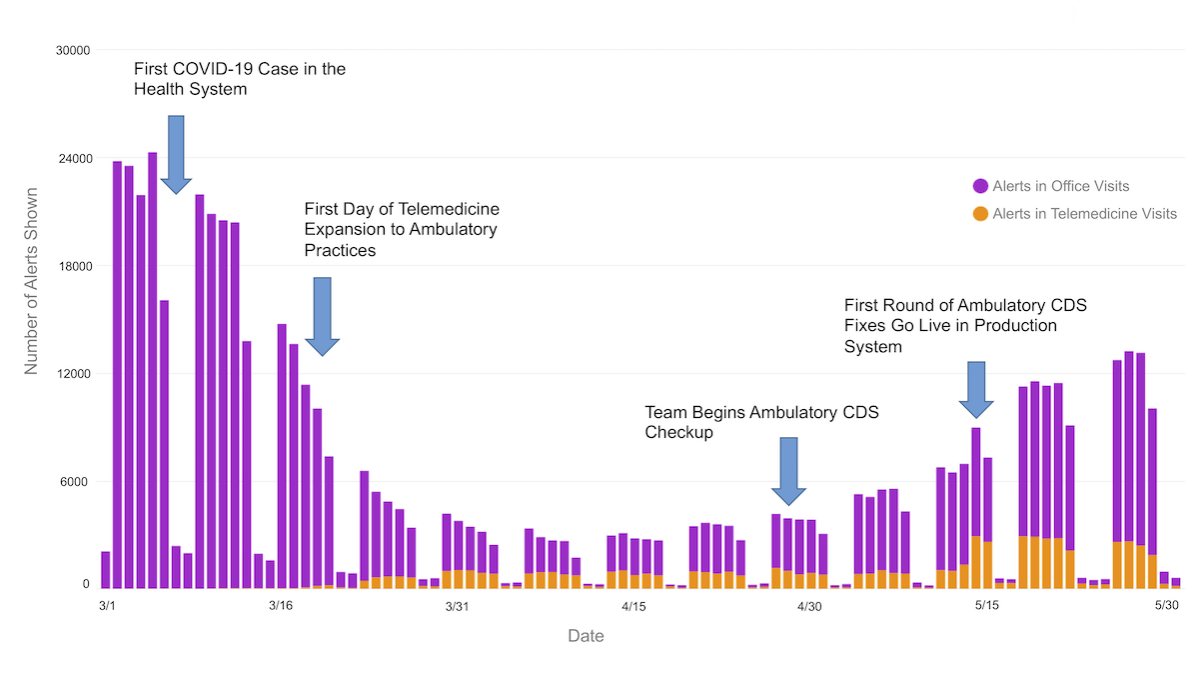
Ambulatory alert firing volumes during the COVID-19 pandemic by date and visit type. CDS: clinical decision support.

Figure 1 shows the overall trend in NYULH daily alert firing volumes at baseline and through the study period.

In total and across ambulatory settings, alert firing volumes were down during the pandemic study period (March 19-May 31, 2020). Still, far fewer alerts were firing in telemedicine encounters (64,938) as compared to office visits (233,636). The relative scarcity of alerts in telemedicine visits was an unexpected finding, even though providers completed more telemedicine visits during this time (244,425 versus 169,651). On a per-encounter basis, during the pandemic, clinicians were shown more than five times as many alerts in office visits (1.37 alerts per encounter) as they were in telemedicine video visits (0.26 alerts per encounter).

**Figure 2:**
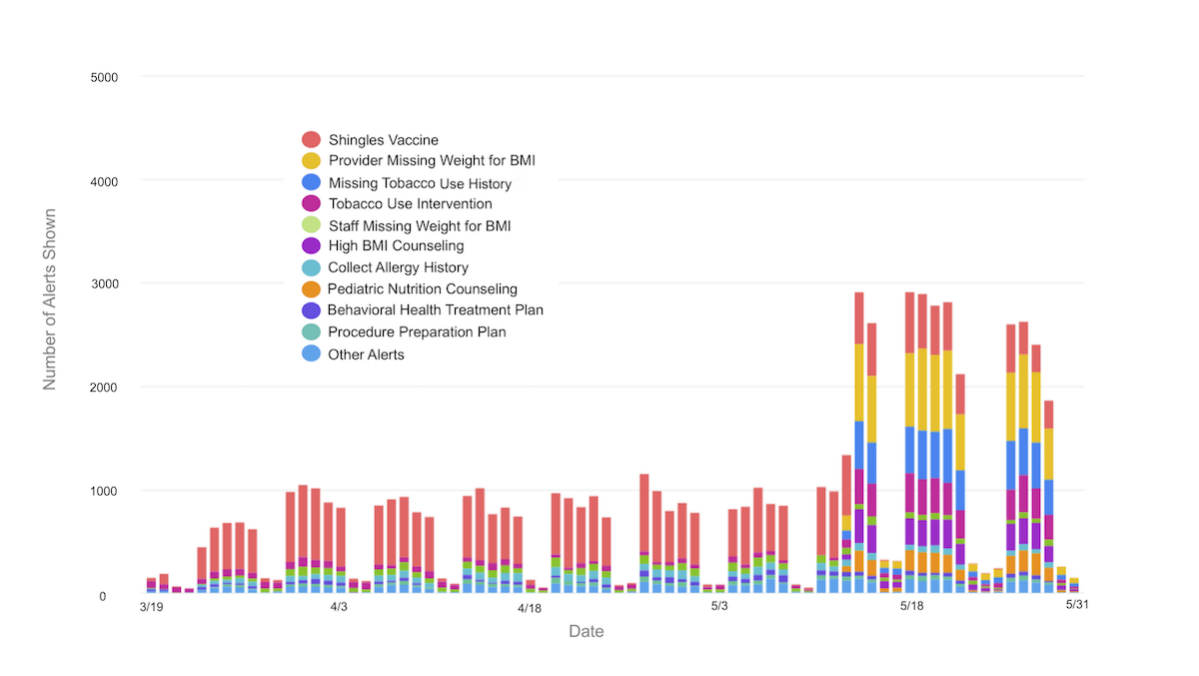
Telemedicine alert firing volumes during the COVID-19 pandemic by date and alert type.

We also compared per-encounter alert firing volumes for each alert in two contexts: telemedicine and office visits. Observing for differences in per-encounter firing volumes in these two settings allowed us to quickly identify malfunctioning alerts that were not firing in a telemedicine setting. We noticed that 10 of our top 40 alerts by volume were not firing appropriately in telemedicine encounters. Further investigation revealed that ambulatory alerts restricted by encounter types were often not firing as expected, while other alerts restricted by practice location or provider specialty were performing well. Clinical informaticists and operational leaders reviewed the list of alerts that were not firing and validated that they were appropriate for the telemedicine encounters. The reconfiguration of these alerts to include telemedicine encounter types went live in the production system on May 14. Figure 2 shows the impact on the overall daily alert volume and diversity of clinical alerts in telemedicine.

### Evaluating Ambulatory Alert Action Taken Rates After the COVID-19 Telemedicine Expansion

To understand whether providers were interacting with alerts displayed in the context of telemedicine encounters at the same rates as during office visits, we looked at the action taken rates in these two clinical contexts during the same study period (March 19-May 31). Table 1 contains the top 5 provider-facing alerts by volume and compares action taken rates for these same alerts displayed in telemedicine and in-person office visits. We found that there were statistically significant differences in the action taken rate in 3 of the top 5 alerts, and in these same 3 alerts, providers were less likely to take action in telemedicine encounters when compared to office visits.

**Table 1:** Action taken rates for the top 5 provider-facing alerts by volume.

Alerts	Telemedicine, n/N (%)	Office visit, n/N (%)	*P* value
Shingles vaccine	1032/26,458 (3.9%)	1576/25,011 (6.3%)	<.001
High BMI counseling	431/3102 (13.9%)	9618/75,144 (12.8%)	.07
Provider missing weight for BMI	24/8101 (0.3%)	21/10,572 (0.2%)	.19
Tobacco use intervention	296/6441 (4.6%)	1543/15,281 (10.1%)	<.001
Pediatric nutrition counseling	85/2139 (4.0%)	517/6381 (8.1%)	<.001

Cumulatively, from March 19-May 31, a total of 64,938 alerts fired in telemedicine encounters, with clinicians taking action on 3257 of those alerts, for an action taken rate of 5.3% (3257/64,938). By comparison, 233,636 alerts fired in office visits, with clinicians taking action on 19,427 alerts, for an action taken rate of 8.3% (19,427/233,636). Although analyses of this type are subject to confounding factors, the superior performance of alerts in office encounters is not surprising. These alerts went through years of iterative improvements specifically for the office setting. Our clinical assessment was that opportunities exist to optimize at least some of these alerts to perform better during virtual visits.

### Clinical Evaluation of Ambulatory Alerts After the Telemedicine Expansion Using the 5 Rights of CDS

Based on the analysis described above, we were able to prioritize alerts for review using the following methodology. For each alert, we calculated the difference in the per-encounter firing rate between telemedicine and office encounters and the difference in the action taken rate in these two settings. We prioritized for review the alerts with the most significant differentials.

During the clinical workflow review, our informaticists reflected on the 5 rights of CDS (the right information, to the right person, in the right intervention format, through the right channel, at the right time in the workflow) [21,22]. Physician informaticists evaluated each alert, looking for opportunities to optimize the alert for telemedicine video visits. As an example of our approach, Table 2 summarizes findings for 4 alerts prioritized for clinical review. Textbox 1 details common overall themes from the informaticist review of multiple alerts.

**Table 2:** Clinical informaticist review of the 5 rights of clinical decision support as applied to NYU Langone Health alerts firing in telemedicine visits.

Alerts	Right time?	Right information?	Right person?	Right format?	Right channel?
Shingles vaccine	Vaccines cannot be given virtually. Telemedicine is only the right time if guidance is for the patient to follow up at the pharmacy or office	Should include a link to shingles vaccine administration locator for available locations that have the vaccine in stock	Yes	Yes	Yes
High BMI counseling	Yes, but alerts not firing without weight being entered	Yes	Yes	Yes	Yes
Provider missing weight for BMI	No, once the video encounter starts, it is already too late. Weight should be collected before the encounter	Yes	Alert should go to patient or office staff	Yes	Consider patient-facing alert through portal
Tobacco use intervention	No, not showing up at the right time in the workflow without staff documenting social history before the provider	Yes	Support staff should be encouraged to virtually room the patient and collect history	Consider adding an interruptive alert after provider enters tobacco use history	Yes

**Themes from clinical informaticist review of NYU Langone Health alerts with discrepant firing rates or action taken rates in telemedicine and office visits.**

Theme 1: Telemedicine visits may have different workflows than office visits, and some alerts developed for the office may not be appearing at the optimal time in the telemedicine workflow.

- Alerts that appear to providers when they enter the encounter during office visits may not appear in a telemedicine encounter until later in the visit.
- These alerts are triggered by clinical data (eg, history, medical problems, vitals, medications) that are usually entered in the office by support staff before the provider sees the patient.
- Without support staff rooming the patient during a telemedicine visit, the alert does not appear until later, when the provider enters this data.
- Noninterruptive alerts are likely to be missed at this later time.

Theme 2: Missing clinical data is a common reason for decreased alert firing rates seen in telemedicine visits.

- Data like vital signs and point of care testing may not be available at the time of the telemedicine visit, and alerts dependent on this data may not fire.
- Without the full care team (eg, medical assistant, nurse, nutritionist, physician extender) contributing to the data collection, reason for visit, medical history, surgical history, social history, medications, and problem list may not be complete.

Theme 3: Remote patient monitoring (RPM) and patient-reported clinical data entered through the portal should have a role in replacing data collection usually completed in the office by a medical assistant or registered nurse.

- The current RPM approach is to collect data between visits. Operational and technical changes will need to be made to optimize RPM for collection on the day of the encounter. This encounter-level data is necessary clinically and would also be available to trigger alerts.
- As patients enter the video visit through the patient portal, there is an opportunity to enter their own clinical data.

Theme 4: When firing rates are down because clinical data is not available, consider workflows where office staff collect data before the provider enters the virtual visit.

- Depending on the need, staff could reach out to patients before or on the day of the visit.
- This strategy would be well paired with RPM and staff playing the role of “virtually rooming” the patient and supporting patient adoption and proper use of remote monitoring.

### Review of Optimization Opportunities Through Existing Governance Structures

At NYULH, we have a multistakeholder CDS governance structure that oversees the CDS life cycle from the initial request to subsequent post–go-live intervention monitoring. Before the COVID-19 pandemic, alert review was conducted on an ad hoc basis. We have now migrated our CDS inventory from an Excel spreadsheet (Microsoft Corp) to a comprehensive knowledge management platform using Collibra’s Data Governance Platform. CDS leadership can initiate automated workflows that send operational owners a message and link to review the CDS metadata and firing rate and document their operational review of CDS. The CDS committee can track these reviews. In parallel, we have made plans to give our operational teams access to Epic’s BPA data model in Slicer Dicer, Epic’s self-service analytics platform. Consequently, the CDS committee and informatics community can more rapidly understand firing rate characteristics to improve the alerts.

With this infrastructure in place, we are currently in the beginning stages of systematically reviewing all CDS interventions, prioritizing ones with high-volume/high-override rates. As we lay the groundwork for success, some early insights include the following: (1) Support from the executive leadership of ambulatory care practices has been particularly critical, even more so than in traditional CDS improvement initiatives, as the next steps involve new operational processes for RPM in virtual care and the changing role of support staff in this context. (2) The first principle of our ambulatory CDS governance is to “avoid interruption of care whenever possible.” Historically, 98.5% of our ambulatory alerts have been noninterruptive. Our CDS stakeholders requested a subgroup analysis of the 2.5% of interruptive ambulatory alerts that fired during the pandemic period; we found that, among interruptive ambulatory alerts, the action taken rate was higher in telemedicine visits (40.5%, 1194/2949) when compared with office visits (29.4%, 691/2370; *P*<.001). These findings were surprising and warrant further study and review; it is possible that in telemedicine encounters, with providers being more immersed in the system, modal alerts are comparatively more effective. The role of changing the alert format for alerts not performing well in telemedicine will likely be an ongoing point of discussion at our CDS governance committee meetings.

## Discussion

### Principal Findings

In this report, we present a framework used to evaluate the impact of telemedicine expansion on our ambulatory CDS program. Based on our findings, we would advocate for other organizations to consider performing their own targeted ambulatory CDS checkup. We provide several vital themes that institutions can target when conducting their own evaluations of CDS in ambulatory telemedicine.

The strength of our approach is in its practical nature, using data that is readily available to prioritize rapid clinical review of CDS alerts most in need of intervention. The weakness may be in its narrow focus. A review of published CDS malfunction taxonomies [23] reveals that the majority of described alert malfunction types may not be discovered using our methodology. We have focused exclusively on best practice advisory alerts, but medication alerts, order sets, documentation templates, and other CDS features should also be re-examined with the shift to telemedicine. There is much work still to be done.

With limitations acknowledged, in a short amount of time, we were able to identify and fix significant CDS malfunctions, recognize alerts in need of optimization, and generate ideas for improving the performance of those alerts. On July 1, 2020, NCQA released “a sweeping set of adjustments to 40 of its widely-used Healthcare Effectiveness Data and Information Set (HEDIS) measures – in support of health plans, clinicians and patients who rely on telehealth services in record numbers as a result of the disruption brought on by the COVID-19 pandemic” [24]. Changes in the HEDIS measures will promote further conversations about quality measurement in telehealth, and will soon lead to increased attention paid to the performance of CDS in this context.

### Conclusion

To our knowledge, this is the first description of how the expansion of telemedicine in response to COVID-19 impacted ambulatory CDS. The COVID-19 pandemic presents many new challenges for the management of chronic diseases. We have demonstrated that an ambulatory CDS checkup focused on telemedicine can positively impact the provision of preventative and chronic care. Our practical framework for reviewing CDS in light of the telemedicine expansion helped identify significant CDS malfunctions and important optimization opportunities.

## Abbreviations

CDS: clinical decision support
EHR: electronic health record
HEDIS: Healthcare Effectiveness Data and Information Set
NYULH: NYU Langone Health
RPM: remote patient monitoring
